# Cross‐country comparisons in health price growth over time

**DOI:** 10.1111/1475-6773.14295

**Published:** 2024-03-07

**Authors:** Irene Papanicolas, Jonathan Cylus, Luca Lorenzoni

**Affiliations:** ^1^ Center for Health System Sustainability (CHeSS) Brown University School of Public Health Providence Rhode Island USA; ^2^ Department of Health Policy London School of Economics London UK; ^3^ European Observatory for Health Systems and Policies London United Kingdom; ^4^ World Health Organization Barcelona Office for Health Systems Financing Barcelona Spain; ^5^ Health Services Research and Policy Department London School of Hygiene and Tropical Medicine London UK; ^6^ Barcelona Institute for Global Health (ISGlobal) Barcelona Spain; ^7^ Organization for Economic Co‐operation and Development Paris France

**Keywords:** cost‐containment, deflators, expenditures, health systems, inflation, international comparisons, prices

## Abstract

**Objective:**

To examine how the United States compares in terms of health price growth relative to four other countries ‐ Australia, Canada, France, and the Netherlands.

**Data Sources and Study Setting:**

Secondary data on health expenditure were extracted from international and national agencies spanning the years 2000–2020.

**Study Design:**

International price indices specific to health were constructed using available international expenditure data and compared to existing health‐specific national and general international price indices.

**Data Collection/Extraction Methods:**

Health expenditure data were extracted from the Organization for Economic Cooperation and Development (OECD) database. We obtained a time series of health price indices from the national agencies in each of the study countries.

**Principal Findings:**

We find meaningful variation across countries in the rate at which health prices grow relative to general prices. The United States had the highest cumulative health price growth compared to general price growth over the years 2000–2020 at 14%, followed by Canada and the Netherlands. Unlike the other study countries, health prices in France grew consistently in line with general prices. Price growth for health care paid for by public funds and households grew at different rates across countries, where price growth was higher for public payers. US households faced the greatest mean annual price growth.

**Conclusions:**

The choice of price index has major implications for comparative analysis. Despite their widespread use internationally, general price indices likely underestimate the contribution of price growth to overall health expenditure growth. We find that in addition to its reputation for having high health price levels compared to other high‐income countries, the United States also faces health price growth for goods and services paid for by government and households in excess of general price growth. Furthermore, US households are exposed to greater health price growth than households in comparator countries.

**What is known on this topic**

- US prices levels for health care are higher than other high‐income countries.
- US health prices tend to grow faster than general economy prices.
- Current methods do not allow for comparisons of health price growth across countries.

**What this study adds**

- Using publicly available international health expenditure data this study constructs health price indices that can be used to examine health price growth across countries.
- The use of health‐specific price indices has major implications for comparative analysis and shows different rates of health price growth across countries.
- The United States faces higher health price growth for goods and services paid for by government and households in excess of general price growth as compared to Australia, Canada, France, and the Netherlands.

## 1 INTRODUCTION

The United States spends considerably more on health care than any other country in the world. A large comparative literature has been devoted to understanding the factors behind high US health spending, generally concluding that high levels of spending in the United States are primarily attributable to higher prices paid for care relative to other countries.^1^, ^2^, ^3^ Most of the literature arrives at this conclusion by either directly comparing price *levels* across countries or by comparing the volume of care delivered at one point in time and finding negligible cross‐country variability in the amount of care provided per person.^1^, ^2^, ^3^, ^4^ Yet the differences in price levels across countries tell us nothing about how changes in prices influence expenditure trends over time. Indeed, there is little literature examining cross‐country variations in price and volume *growth*. As high‐income countries experience a period of economy‐wide price growth not seen in decades—with inflation in 2022 reaching a high of 8.5%, and 10.6%, in the United States and the Euro area, respectively^5^, ^6^—it has become increasingly important to better understand the rate of price growth in the health sector.

To compare health expenditures over time across countries, analysts often deflate health expenditure levels by removing the price component; the remaining measure, reflecting the volume of care provided, is referred to as real expenditure levels. There are currently three types of price indices commonly used to deflate health expenditures: economy‐wide price indices, such as the gross domestic product (GDP) price index, price indices that cover all goods and services consumed by households, such as the actual individual consumption (AIC) price index, and national health deflators, such as those produced by the Center for Medicare and Medicaid Services (CMS), the Bureau of Economic Analysis (BEA) and the Bureau of Labor Statistics (BLS) in the United States.^7^ Although they are widely used to deflate health expenditures for international comparisons, the GDP and AIC price indices capture changes in prices overall—not changes in healthcare‐specific prices. Given that health care is often provided by non‐market producers such as governments (where prices may be imperfect signals of market value), healthcare prices are likely to differ markedly from economy‐wide or AIC prices. Furthermore, these price indices do not account for differences in price trends by financing scheme, an assumption which implies that different purchasers of health care, such as governments and households, face the same growth in prices.

National health price indices, such as those routinely used to measure health inflation in the United States, have been developed to better allow countries to explore health price growth over time. However, few countries produce national health price indices, and the methods used across countries to measure growth in health prices differ in their formulas, scope, and sources of data, limiting the potential for cross‐country comparability in terms of price growth, and as a result, also in terms of real expenditure on health. If we use national health deflators to compare trends in health price inflation across countries over time, we cannot determine if observed differences across countries are because of differences in a health system's ability to control health price growth, or because of differences in the way countries measure health prices and their growth.

A range of factors influence health spending growth through their effects on prices and the volume of care. These factors include economic growth, population aging, technological change, and institutional factors such as health insurance coverage.^8^, ^9^ Evidence from the United States suggests that nearly two‐thirds of growth in health spending is accounted for by national income and the interaction between income and technology.^8^ However the extent to which income and technology influence either health prices or volumes—and ultimately expenditure growth—in different countries will likely differ depending on institutional factors such as health financing mechanisms, insurance markets, and cost containment polices. Without an internationally consistent approach to measure health prices, analysts are limited in the conclusions they can draw about how or why health policies and institutional characteristics affect expenditure growth. This means, for example, that analysts cannot say whether policy interventions that slow expenditure growth are likely to do so through effects on prices or through reductions in the volume of care provided.

This paper explores the approaches currently used to deflate health spending and examines the feasibility and implications of using alternative health price deflators which can be constructed from available international expenditure data. We seek to address the following questions: (1) Using the price deflators currently available, and newly constructed deflators based on national accounts data, what does health price growth look like over the past 20 years across a selection of high‐income countries? (2) How does the growth in health prices over the past 20 years compare to growth in general economy and household consumption prices across countries? (3) Using the different approaches to deflate health expenditures over time, how does real health expenditure growth vary across countries, and how does it vary by type of financing scheme, such as public expenditure versus household out‐of‐pocket expenditures?

## 2 DATA AND METHODS

This paper explores growth in health prices based on analyses of price deflators and expenditure data produced by national and international organizations. One of the tasks in the international comparative analysis of health expenditure time series data is to separate out the part of expenditure growth that stems from a change in quantities or volume of services and goods provided (i.e., output), from the part that stems from price changes in those same goods and services. To obtain estimates of volume growth, analysts can divide the change in health expenditure levels over a period by the change in the prices of the corresponding products. This produces a measure of the change in volume, that is, real health spending. This approach is what national accountants refer to as “deflation.” It should be noted that this is different from what economists call “deflation,” which refers to a period of sustained drop in prices affecting economic agents' expectations.

### 2.1 National price deflators for health care

We identified five countries that produce national price deflators specific to health care: Australia, Canada, France, the Netherlands, and the United States, and obtained time series of health price indices from the respective national agencies in each of these countries for the years 2000–2020 (Appendix S1).^10^, ^11^, ^12^, ^13^ Appendix S1 reviews the national price indices for these countries, illustrating some of the differences in the types of goods and services they account for. For example, some countries include services such as dental care (Australia, France) and eye care (France, the United States) while the Netherlands and the United States also include nursing care. Moreover, some countries produce different indices to deflate private and public expenditures, while others disaggregate prices by different geographical region or by more granular types of health spending.

### 2.2 Alternative methods to measure implicit healthcare price indices

We consider a number of alternative approaches in an effort to indirectly estimate internationally comparable healthcare price indices based on national accounts data provided to international organizations. Countries compile expenditure time series data at current prices and constant prices (i.e., reflecting volume) based on the System of National Accounts framework to measure economic activity across sectors.^14^ To compare spending on market goods and services at constant prices, deflators are typically applied at industry section or sub‐section level or at the level of specific types of output, such as components of consumer price indices or producer price indices. This has the benefit of ensuring that the resultant volume estimates are all in the same price base and that changes in the quality of goods and services are captured in the volume component.

A large part of health spending stems from non‐market transactions for which prices are not directly observed or measurable. As a result, many countries do not produce health‐specific price indices for the purpose of international comparisons. However, countries do report changes in volumes for non‐market sectors using four types of approaches^15^: (1) deflation using an estimate of output prices; (2) deflation using an estimate of input prices; (3) direct output indicators; and (4) direct input indicators. The first two categories are considered “indirect” methods (deflation) as the volume estimate is created “indirectly” through deflation using a chosen price index. The second two categories are considered “direct” methods because the chosen indicator is used to directly capture changes in volume. For certain non‐market output such as health care and education, most of the countries in our study group use the direct output indicators measurement approach (Australia, France, and the Netherlands), Canada uses the direct input indicators measurement approach, while the United States uses the output price deflation approach. For public administration and defense typically no direct output measures are available and deflation using input prices is used. These methodological differences are important to note, as they too can influence the comparability of volume estimates, and ultimately also price estimates across countries.

These volume measures can be used to implicitly measure price growth. So‐called “implicit price deflators” (IPD) are calculated by dividing an expenditure time series by the corresponding time series measured in volume terms; the difference between these series reflects prices. IPDs are calculated at different levels of aggregation—including for gross domestic product (GDP) and actual individual consumption (AIC). The different indices vary in the type of outputs they consider and thus can be used to adjust for price changes for different baskets of goods and services. The AIC IPD covers all goods and services consumed by households, including, for example—in addition to health care—food and beverages, transports, and culture. Therefore, this index reflects price changes in household consumption. The OECD currently uses this index to deflate health expenditures. The GDP IPD is broader and considers all goods and services produced in an economy, including gross fixed formation and net exports. This index reflects price changes across all goods and services produced domestically. This index is used by the World Health Organization (WHO) to deflate health expenditures. Table 1 outlines the differences between these indices in terms of their coverage.

**TABLE 1:** International deflators and their coverage of goods and services.

Acronym	Name	Coverage
GDP	Gross Domestic Product implicit price deflator	It represents the broadest measure of inflation in the domestic economy, reflecting changes in the price of all goods and services that comprise GDP
AIC	Actual Individual Consumption implicit price deflator	It consists of all goods and services actually consumed by individuals, irrespective of whether they were purchased and paid for by households, government, or non‐profit institutions serving households
Government FCE	Individual Final Consumption Expenditure of government implicit price deflator	Government final consumption can be broken down into two distinct groups. The first reflects expenditures for collective consumption (defense, justice, etc.) that benefit the society as a whole. The second, referred to as “individual”, relates to expenditures for individual consumption (health care, housing, education, etc.), incurred by government for the benefit of individual households.
Household FCE – health	Household Final Consumption Expenditure for health implicit price deflator	It represents all out‐of‐pocket expenditure for health of private households

The appropriate use of an index depends on the question being asked. For example, if one is interested in the share of resources devoted to health care relative to other sectors of the economy, one might want to use the GDP deflator. However, if one wanted to better understand the volume/price breakdown of health expenditures over time, a health‐specific deflator would be more appropriate. Using an economy‐wide deflator for this purpose would not allow one to distinguish between differential price growth in health as compared to other sectors.

As a health‐specific deflator does not currently exist for international purposes, we develop an alternative IPD specific to health care to examine the volume/price breakdown of health expenditures across countries. We compare this to the AIC and GDP IPDs, which are the international standard used to compare health expenditures across countries, and to national health‐specific deflators.

We extracted health expenditures at current and constant prices for our study group of countries from the Organization for Economic Cooperation and Development (OECD) database (https://stats.oecd.org). We constructed a new health consumption‐based IPD using two national account series—estimated at current and constant prices—on final consumption expenditure (FCE) based on the System of National Accounts data collection framework^14^: household FCE for health and individual FCE of government (See Table 1). The FCE of government includes spending on health, education, and current expenditure on defense and public administration. Across OECD countries, spending on health represents 15% of total government spending on average. The household FCE for health includes services and products that can only be used in response to a health need. Across OECD countries, the consumption on health goods, on average represents only 5% of household total final consumption. Appendix S2 outlines in more detail the series used to construct IPD for GDP, AIC, and health consumption, and the corresponding national sources that contribute to each series. These are available to extract from the OECD database (https://stats.oecd.org) and produced by national statistical authorities following the system of national accounts collection framework and collected by the OECD via an annual questionnaire.

To construct a health deflator that captures both government and household expenditures, we weighted the IPDs for these two components using the share of health spending financed from public versus private sources, as measured in the System for Health Accounts (a framework that offers guidelines for reporting internationally comparable measures of health expenditure by financing source, provider and type of service).^16^ For the United States, we assume that spending by private health insurance and other payers will have price trends similar to the combination of out of pocket (OOP) payments and government. Prior work has shown that for the hospital service component, private insurance prices grow faster than prices in public programs and slower than the consumer price index.^7^ Of note, this approach we use to construct the consumption‐based IPD is similar to the approach used by Statistics Canada to construct its national health price index.^11^

**FIGURE 1:**
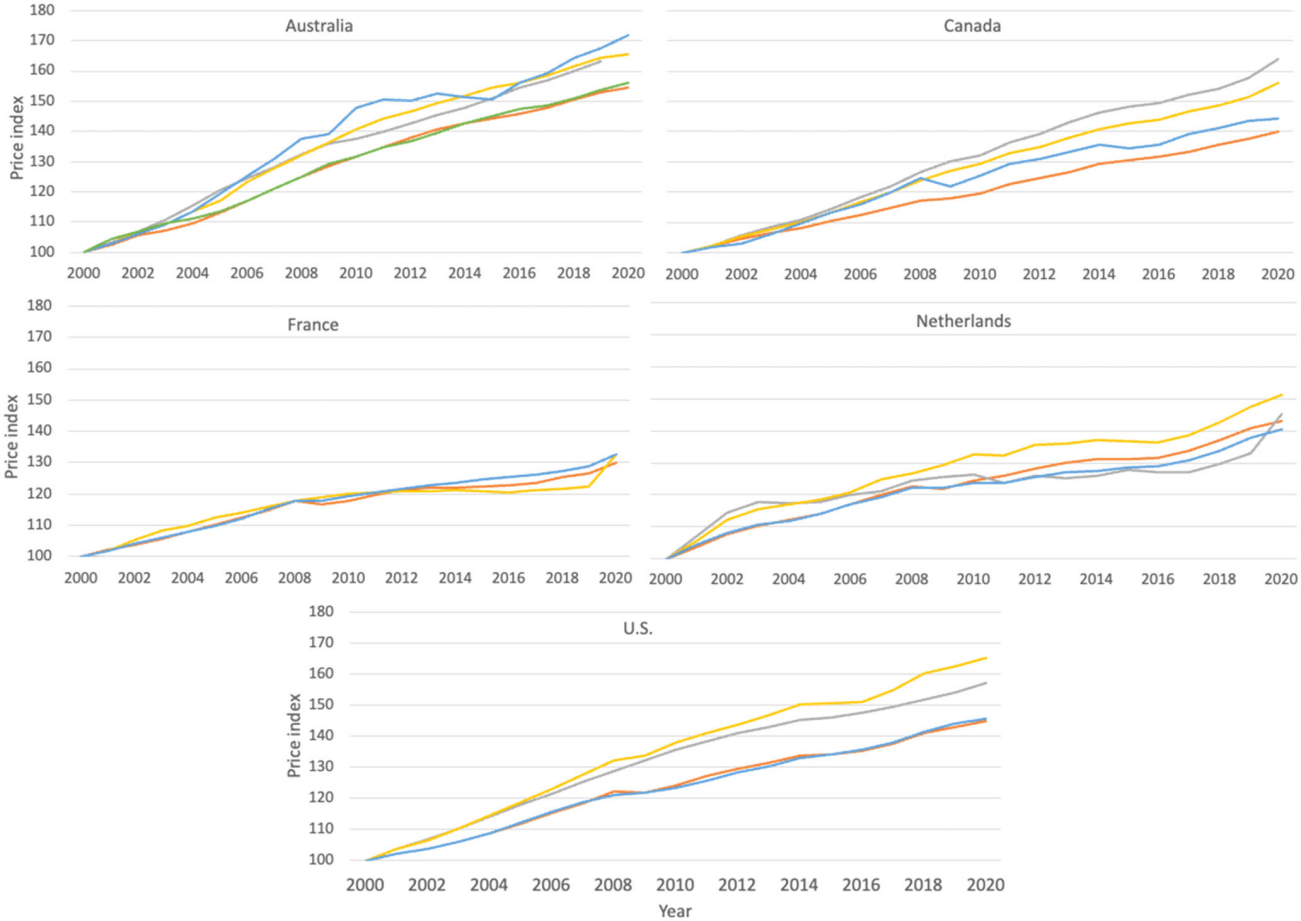
Trends in health care prices across countries using available deflators (AIC, GDP, national health, health consumption). AIC refers to the actual individual consumption deflator. National health refers to each country's own national health deflator. Health consumption refers to the alternative health deflator constructed by the authors. GDP refers to the gross domestic product deflator. For Australia, we also use a Gross National Expenditures (GNE) deflator to account for the export mining boom during the study period. For France, the points for the years 2000–2010 are estimated by extrapolating the national health price index backward using its average growth rate observed between 2010 and 2015. *Source*: Authors analysis of national expenditure accounts extracted from the Organization for Economic Cooperation and Development (OECD) database and National Statistical Agencies.

Using the AIC and GDP IPDs, the national health‐specific deflators, and our newly constructed consumption‐based health‐specific deflator—we compare price and volume growth over the period 2000–2020. For Australia, we use a Gross National Expenditures (GNE) deflator in place of the GDP deflator, as there was a large mining boom^17^ leading to increased income from exports which affects the GDP deflator series. For France, the national health price index was available from 2010 onwards. For illustrative purposes for Figure 1, we extrapolated backward to 2000 using the index's average growth rate observed between 2010 and 2015.

Next, we explore differences in the relative growth in health prices and general prices by calculating the ratio of cumulative growth between our newly constructed consumption‐based health‐specific index to the AIC consumption‐based index measuring overall prices (“excess” health price growth).

Finally, we examine trends in per capita real health expenditure growth by type of financing, namely expenditure from government schemes and compulsory health insurance (which we call “public”), and household out‐of‐pocket and voluntary health insurance expenditures (which we call “households”). To do this we deflate expenditures from government schemes and compulsory health insurance (public) on health care using the FCE government IPD and household out‐of‐pocket and voluntary health insurance (households) expenditures by the IPD household FCE for health. For the United States, we deflate expenditure from government schemes on health care (48% of total spending according to the National Health Expenditure Accounts^13^) using the FCE government IPD and the household OOP expenditure on health (10% of total spending) using the IPD household FCE for health. We do not examine trends in spending by private health insurance (29% of total health spending) and other payers (13% of total health spending).

## 3 RESULTS

Figure 1 shows the growth in prices over the period 2000–2020 across the five countries as measured by the different price indices. For ease of interpretation, the prices are indexed to 100 in each country at the year 2000. The actual price levels across indices and countries are however different. Comparative price levels (CPL) for health are estimated to be 5% higher than CPL for AIC on average across OECD countries.^18^ The United States shows a CPL for health 43% higher than OECD average, whereas for France the CPL for health is 30% lower than OECD average.^19^

The indices reveal different trends in price growth across all countries, with no single price index growing consistently slower or faster across all countries.

Except for the Netherlands, the two health‐specific indices (national and consumption based) and the two general indices (GDP and AIC) track more closely together, respectively. In Australia, Canada, and the United States, the health indices show faster price growth over time than the general indices, while in France, health prices grow slower than general prices. Across all indices, Australia has the fastest health price growth over the period and France the slowest. The United States and Canada have the biggest gap between the different price indices by the end of the period in the study, with the health‐specific indices growing faster than the general price indices. The largest gap, for both countries, exceeds 20 percentage points difference, if within‐country price levels were equivalent in 2000.

Next, we use the four different price indices to deflate health spending across countries, allowing us to examine how much of health spending growth is related to volume versus price growth over the study period. Table 2 reports the average annual growth rate of per capita health spending at current prices, and at constant prices, as calculated using each deflator. For all countries, the use of the AIC deflator suggests greater volume growth, and a lower contribution of price growth, to overall health expenditures as compared to the health‐specific deflators. The estimates using the GDP deflator are closer to the national health‐specific deflators for Australia and Canada, but for the other countries are similar to the estimates produced by the AIC deflator. The results obtained using the health‐specific consumption‐based deflator are more closely in line with the results obtained using the national health‐specific deflators, except in the case of France where the results were similar across all price indices.

**TABLE 2:** Mean annual percent growth rate in per capita health spending at current prices and at constant prices using international and national price deflators (2000–2020).

	Mean annual % growth rate				
		At constant prices			
	At current prices	GDP	AIC	Health consumption	National
Australia	5.7	2.9	3.4	3.0	3.0
Canada	4.7	2.8	3.0	2.4	2.2
France	2.9	1.5	1.6	1.5	1.7*
The Netherlands	4.3	2.6	2.5	2.2	2.4
The United States	4.9	3.0	3.0	2.2	2.6

Source: Authors analysis of national expenditure accounts extracted from the Organization for Economic Cooperation and Development (OECD) database and National Statistical Agencies.

*Note*: GDP refers to the Gross Domestic Product deflator, AIC refers to the Actual Individual Consumption deflator. Health consumption refers to the alternative health deflator constructed by the authors. National refers to each country's own national health deflator. *The national health price index for France began in 2010.

**FIGURE 2:**
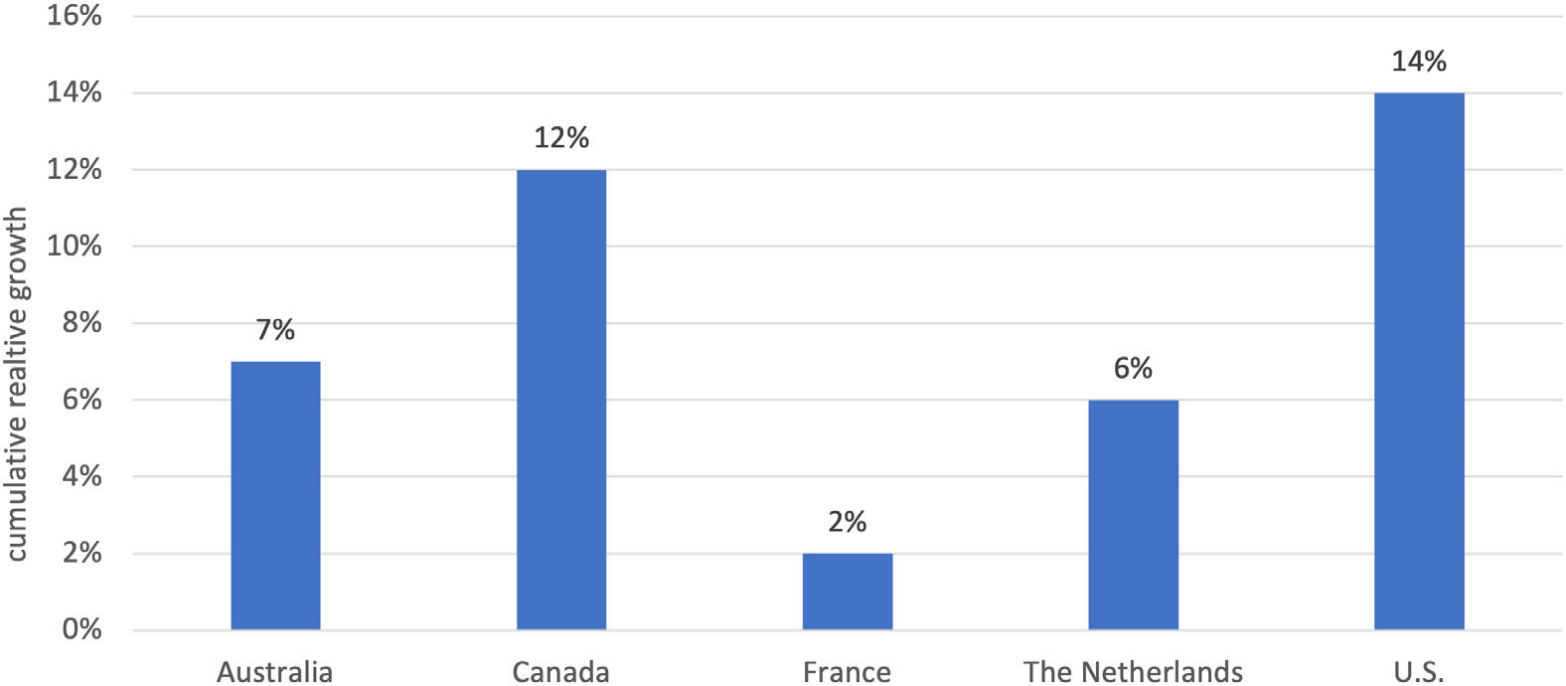
Cumulative relative growth of health prices to general prices between 2000 and 2020 (Authors' health consumption price index/AIC price index). AIC refers to the Actual Individual Consumption deflator. Health consumption refers to the alternative health price deflator constructed by the authors. *Source*: Authors analysis of national expenditure accounts extracted from the Organization for Economic Cooperation and Development (OECD) database and National Statistical Agencies.

When using the health‐specific consumption‐based deflator, price increases explain a larger share of health spending growth in the United States compared to the other countries in the study (55% vs. 48%). By looking at the ratio of the consumption‐based health‐specific index to the consumption‐based general price index (AIC) over the study period (Figure 2), we find that the United States shows the highest relative cumulative “excess” growth in health prices over the study period (14%), followed by Canada (12%). Australia and the Netherlands also exhibit higher cumulative health price growth over the study period compared to general price growth, although slightly lower (7%–6%) than the first cluster of countries. Finally, France shows the lowest cumulative price growth, with health prices growing only slightly above general prices (2%).

**FIGURE 3:**
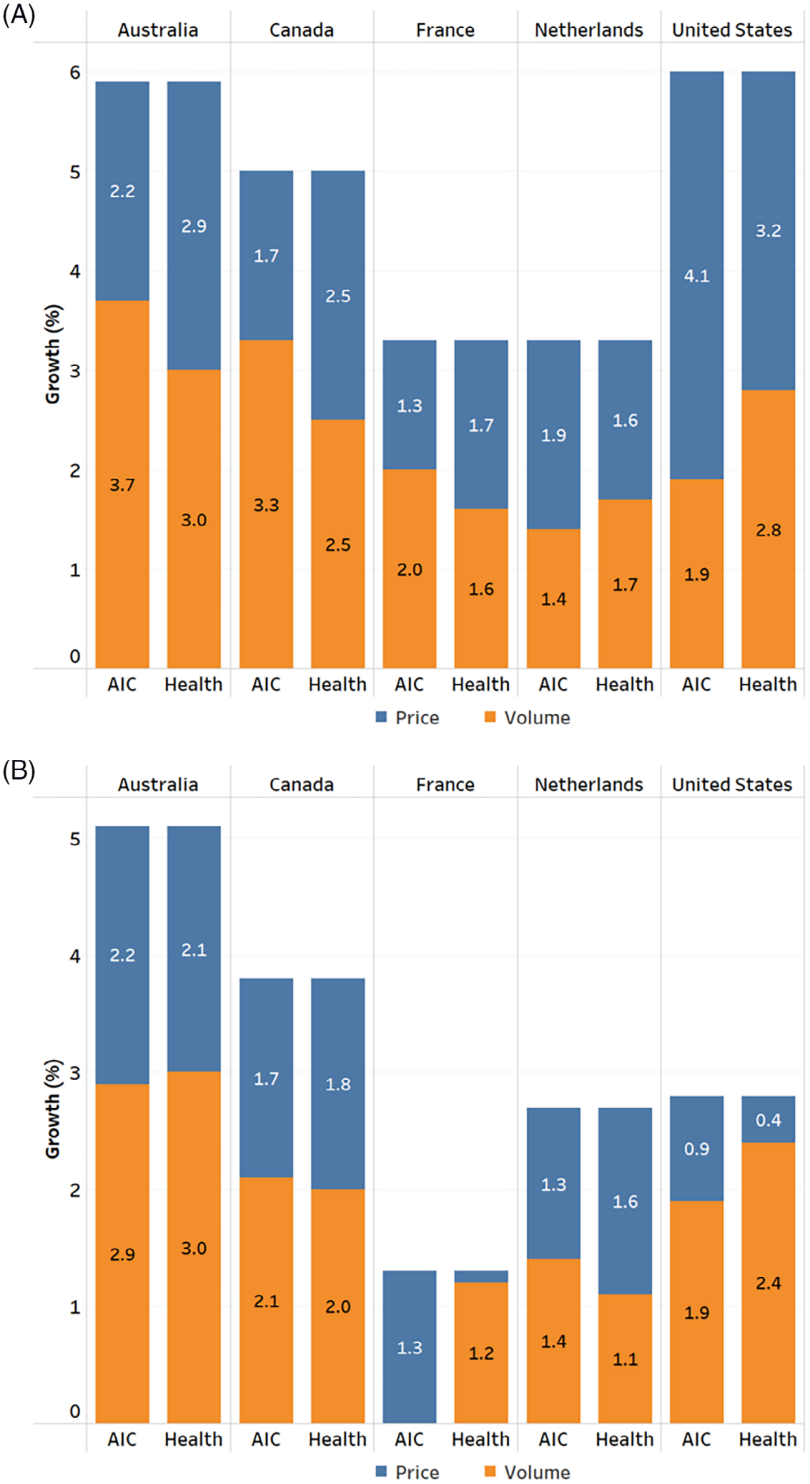
(A) Mean annual percent growth rate in health prices and per capita health volumes using AIC and health consumption deflators by public payer, 2000–2020. *Source*: Authors analysis of national expenditure accounts extracted from the Organization for Economic Cooperation and Development (OECD) database. (B) Mean annual percent growth rate in health prices and per capita health volumes using AIC and health consumption deflators by households, 2000–2020. AIC refers to the Actual Individual Consumption deflator. Health refers to the health consumption deflator constructed by the authors. The category “public payer” represents the share of expenditures classified as government or compulsory health insurance.AIC refers to the Actual Individual Consumption deflator. Health refers to the health consumption deflator constructed by the authors. The category “households” represents the share of spending on out‐of‐pocket payments and voluntary health insurance, apart from the United States where it only represents out‐of‐pocket payments.

We then compared the average annual growth rate of per capita health spending from public funds and from households separately, using either the AIC deflator for both types of expenditure or the financing scheme‐specific deflators described in Table 1. For all countries the use of a scheme‐specific deflator results in lower average annual growth in real per capita spending from public sources as compared to the use of the AIC deflator (Figure 3A).

When applying the sector‐specific deflator to household expenditures we find higher average annual growth in real per capita spending for Australia, France, and the Netherlands and lower annual growth in real per capita spending for Canada and the United States (Figure 3B). Notably, when we apply the sector‐specific deflator we find that US households face the greatest mean annual price growth rate across all countries. The results for France also differ considerably when using the household‐specific price deflator. The average annual growth rate of the price index for households is 1.3% based on the AIC deflator and 0.1% for households based on the FCE for health. This suggests a growth rate for real household spending on health of 1.2% when the household‐specific deflator is used (instead of no real growth when the AIC deflator is used).

The comparisons between Figure 3A,B also illustrate the average annual price growth over the study period faced by public payers and households. In all countries, price increases were greater or equal to in publicly funded health care than for households. In addition, the difference in the average annual growth rate in health spending between public payers and households is smaller when using the sector‐specific deflators as compared to when using the AIC deflators.

## 4 DISCUSSION

In this study, we compare the growth in health prices over the period 2000–2020 in five high‐income countries—Australia, Canada, France, the Netherlands, and the United States using an international health consumption‐based deflator constructed from existing national accounts. We show that our deflator performs comparably to national health deflators, and illustrate that there is meaningful variation across countries in the rate at which health prices grow relative to general prices, which is not captured when economy‐wide price indices are used to deflate health expenditures. Our findings suggest that the United States had the highest cumulative health price growth compared to general price growth over the study period, at 14%, closely followed by Canada (13%). Unlike the other study countries, health prices in France overall grew most closely in line with general prices (2%). Our results also suggest that price growth for health care purchased by public funds and households grew at different rates across countries, where price growth was higher for public payers. We also found variability with regard to the price growth that households bear across the study countries, with US households facing the greatest mean annual price growth.

These findings have important implications for policy makers interested in understanding how health prices grow over time, and in relation to general prices. First, our results demonstrate the importance of developing comparable health‐specific price deflators to analyze the growth of health expenditures. Cross‐country variation in health expenditure growth could be the result of differences in the growth of prices of goods and services or differences in the growth of volume of care, or a mix of both. Disaggregating health spending into volume and price measures helps policy makers better understand how these factors evolve and help them decide what policy responses should be put in place to address health spending trends. Such policies may differ if, for example, a country's high healthcare spending growth is due to relatively high growth in the volume of goods and services consumed or to the relatively high growth in prices paid for those goods or services. Thus, it is critical that the price index used to disaggregate health spending into volume and price measures accurately reflect healthcare price changes.

While the international standard has been to use GDP indices or household consumption indices to deflate health spending due to their widespread availability, using these indices to proxy health price growth limits our ability to compare the growth in *health* prices to general prices. As a result, in this paper, we examine the feasibility and implications of using an alternative consumption‐based *health* price deflator that can be constructed using internationally available data (based on household FCE for health and individual FCE of government). Our results suggest that the health‐specific deflator seems to be in line with the change in volume estimated at national level using price indices computed for health spending for those countries where national health price data are available.

Our results using the alternative consumption‐based health price deflator on a small sample of five high‐income countries also demonstrate that the growth of health prices in relation to general prices varies across countries. This suggests that the policies used by health systems to regulate prices (both in health and the general economy) may have differential effects on health price growth and that there are important potential areas for cross‐country learning. There are many differences in the ways in which health prices are regulated and set across our study countries that may contribute to the trends we observe. We observed the greatest health price growth in Australia and the United States. However, the Australian health price growth was more in line with general price growth than the United States, as shown by the smaller gap between the health price growth estimates using the health‐specific and economy‐wide deflators. Higher general inflation in the study period in Australia is well documented.^17^ In the United States, and in Canada, however, the growth in health price indices deviated more from that of the general price indices, suggesting that there are potential health sector‐specific drivers of price growth in these countries.

While in most countries health price growth outpaced that of general prices this was not the case in France. France had both the lowest health and general price growth across the countries, and health prices grew only slightly faster than general prices. While there are many factors that likely contribute to lower overall inflation in France, there are also notable health‐specific policies aimed at keeping health price growth under control. Specifically, France sets a projected ceiling on health spending each year, which limits both price and volume growth.^20^

For the United States, the Netherlands may be the most interesting comparator of the study countries, given the similarities in terms of health system design. The Netherlands also has competing insurers, and while health prices grew faster than general prices the gap is smaller than in the United States. Moreover, in the Netherlands mean annual price growth faced by households is lower than price growth in the compulsory insurance market. There are many potential explanations for why this may be the case: the Netherlands relies sparingly on out‐of‐pocket payments to finance health care, has considerable price regulation across all care settings, and sets a relatively low deductible capping user charges.^21^ More work is necessary to better understand how these and other national policies influence health price growth, possibly drawing on a wider set of countries.

Our analysis examining price growth trends separately for different financing schemes within countries offers further insights. Across all countries, price growth has been comparatively higher for care paid for by public schemes than care paid for out of pocket by households. Our results also show that US households are more exposed to health price growth than households in comparator countries. Applying the financing scheme‐specific deflator to France, we observe that health prices for households have grown slower than general prices, implying that growth in health expenditures over the study period is largely attributable to growth in the volume of care consumed by households.

There is scope for more work in this area. Implicit price deflators for household FCE could also be calculated for medical products, outpatient services, and hospital services for most OECD countries. As the use of policy to regulate price and volume in these care settings differs both within and across countries, future work should also focus on the feasibility and impact of developing specific deflators by care setting. This should help further improve current approaches, which rely on AIC or GDP deflators to disentangle the contributions of price and volume components for each aggregate spending category.

We believe this study makes important contributions to the literature on both health prices and health system comparisons. Most comparative studies that have examined health prices either do so by comparing prices for specific health goods or services at one point in time, or by comparing price levels for a basket of goods and services.^4^, ^22^, ^23^, ^24^ Within countries, there is some literature examining health prices over time and how they compare to general prices, most notably in the United States.^7^, ^25^ However, this has not been extended to understand whether these trends are similar in other countries. Our study adds to this literature by examining health price *growth* across countries. We contribute to this area by both proposing a new approach to deflate health expenditures across countries, but also by producing comparable estimates of health price growth across a subset of high‐income countries.

Our study also contributes to the literature examining the rate of growth of price and volume in health. Notably, most of this literature has been focused on studying the determinants of health expenditure growth overall, such as uptake of new technologies and population demographics.^8^, ^9^, ^26^, ^27^ Our study extends this literature by building on the approaches used to disaggregate trends in health expenditures into price and volume growth across countries. To compare health price and volume growth across countries, the use of comparable health‐specific price deflators is essential as—otherwise—differences in “excess” growth in health prices would be captured in the other factors influencing health spending trends, including economic growth, population aging, and technological change.^8^, ^9^ Importantly, our application of health‐specific deflators to health expenditures demonstrates meaningful variation in price growth across countries, suggesting that short‐term health system design features, such as the approach toward price regulation can affect healthcare price growth.^28^ There is potential for cross‐country analysis to more precisely identify and understand these health system characteristics.

This analysis highlights the current limitations not only in being able to produce comparable estimates of health price growth across countries but also the limitations for deflating health spending to estimate a temporal volume versus price breakdown and calculate real health spending levels and growth. We find a large variation in health price growth within countries over time depending on the choice of price index. It is critical that the price index used to deflate spending accurately reflects health price changes. Even if we acknowledge that more efforts to create internationally comparable health price data are needed to further help analysts and policymakers to better identify and understand potential mechanisms to effectively slow health price growth in the United States and other countries, the new approach to deflate health expenditures across countries proposed in this paper represents an important improvement in this direction.

Our study has limitations. First, the timeliness and coverage of the national accounts time series data are imperfect for our purposes. In particular, the government FCE data encompass all government spending, including for goods and services whose prices are likely to differ from prices paid by government for health care. More work needs to be done in the future to improve data collection and reporting. Second, as noted above, the approach used to measure volume for non‐market services varies by country. National Statistical Institutes (NSI) use different methodologies to estimate government FCE output and to adjust for quality changes of output to compile national accounts data. Third, the household spending component differs for the United States as compared to other countries. In all other study countries household spending represents spending on out‐of‐pocket payments and voluntary health insurance, whereas in the United States it only represents out‐of‐pocket payments. This may limit the comparability of the United States estimates of annual per capita volume growth with the other countries.

## 5 CONCLUSIONS

Using a new approach to deflate health expenditure, this paper shows that in addition to having higher health price levels as compared to other high‐income countries, the United States also faces high health price growth relative to general prices. In addition, US households are more exposed to health price growth than households in comparator countries. Policy makers should examine health system design features in other countries to learn more about how to effectively control health price growth in the United States.

## FUNDING INFORMATION

The research in this article was not funded.

## CONFLICT OF INTEREST STATEMENT

The opinion expressed and arguments employed in the article are solely those of the authors and do not necessarily reflect the official views of the OECD or of its member states.

## Supporting information


**Appendix S1.** National deflators and methodologies used to measure non‐market outputs for health.
**Appendix S2.** Price indices, series, and data sources used to construct implicit price deflators.
