# Supplementary material for: Cross‐country comparisons in health price growth over time
Source: Health Serv Res. 2024 Mar 7;59(6):e14295. doi: 10.1111/1475-6773.14295 (PMC11622263; doi:10.1111/1475-6773.14295)
Supplement: Supplementary file 1 — Appendix S1. National deflators and methodologies used to measure non‐market outputs for health. Appendix S2. Price indices, series, and data sources used to construct implicit price deflators. [file HESR-59-0-s001.docx]

**Cross country comparisons in health price growth over time**

SUPPLEMENTARY APPENDIX

**Appendix 1:** **National deflators and methodologies used to measure non-market outputs for health.**

| **Country** | **Name of Index** | **Producer** | **Coverage** | **Methodology applied** | **Source** |
| --- | --- | --- | --- | --- | --- |
| Australia | Total Health Price Index (THPI) | Australian Institute for Health and Welfare (AIHW) | Includes spending on 15 areas of public and private expenditure: public hospitals, private hospitals, patient transport services, medical services, dental services, community health services, public health, benefit paid phramaceuiticals, all other medications, aids and appliances, administration, reseach, capaital expenditure, medical expenseses tax rebate | Specific deflators for the different categories of spending are applied to current prices from the latest financial year to get constant prices. THE in current prices is then divided by the THE in constant prices to get the THPI deflator. | <https://www.aihw.gov.au/reports/health-welfare-expenditure/health-expenditure-australia-2019-20/contents/about> |
| Canada | Total Health Care Implicit Price Index | Statistics Canada | Includes spending on public and private expenditures in each province and territory. The health component of the Consumer Price Index is used to deflate private-sector health care spending. The implicit price indices (IPIs) for government current expenditure are used to deflate public-sector health care spending. | Public and private expenditures are adjusted separately in each province using the appropriate index. Adjusted values are summed to obtain Canada totals at constant dollar values. | <https://www.cihi.ca/sites/default/files/document/nhex-trends-2021-meth-notes-en.pdf> |
| France | Price Index for Health | Direction de la recherche, des études de l'évaluation et des statistiques (DRESS) | Includes spending on 16 different areas of public and private expenditure: public hospitals, private hospitals, primary care, specialist care, midwife care, dental services, laboratory services, spa treatments, reimbursed phamaceuticals, non-reimbursed pharmaceuitcals, eye care, prostheses, aids and appliances | The price index applied to each area of spending is either an implicit price index (e.g. public hospitals) or a direct price index (e.g. private hospitals, General Practitioners). The total health price index is a weighted average – the weights being the expenditure shares – of the price indices by area of spending. | <https://data.drees.solidarites-sante.gouv.fr/explore/dataset/306_les-comptes-de-la-sante/information/> |
| The Netherlands | Price Index for Health | Statistics Netherlands (Centraal Bureau voor de Statistiek, CBS) | Incudes spending on health broken down by eight different areas of spending/providers: providers of medical specialist care (e.g. hospitals), mental health providers, general practices, dental practices, paramedical practices, nursing care providers, disability care providers, other providers (e.g. preventative care, patient transportation). | For each area of spending/provider a price index is computed in three ways: (1) Direct changes in price are mreasured for each type of service delivered by the provider. (2) A proxy is used to estimate price changes: either maximum prices set by the National Health Authority (e.g. for dentists, GPs and maternity care), or CPI (e.g. for physicotherapists and opticians(. (3) Volume changes are used to indirectly estimate price changes (e.g. number of breast and colon cancer screenings). | StatLine - Zorguitgaven; volume- en prijsontwikkelingen (cbs.nl) |
| U.S. | Personal Health Care Price Index | Centers for Medicare and Medicaid Services | Includes personal health expenditures (out-of-pocket and third-party) only on which include hospital care, phsycians and clinical, other professional services, dental services, other health residential and personal care services, home health care, nursing home care, prescription drugs, other non-durable products, durable medical products. Excludes government administration, net cost of insurance, noncommercial research, and private capital investment. Built from component CPI and PPI components. | A chain-weighted price index is calculated for the various goods and services that account for PHC spending in the national health expenditure. | <https://www.bls.gov/cpi/factsheets/medical-care.htm> |

**Appendix 2: Price indices, series and data sources used to construct implicit price deflators**

| Implicit price deflator | Component | Series used to construct the implicit price deflator | Source - Australia | Source - Canada | Source - France | Source – the Netherlands | Source – United States |
| --- | --- | --- | --- | --- | --- | --- | --- |
| GDP |  | Annual National Accounts of OECD countries. Gross Domestic Product – expenditure approach - at current prices and at constant prices (base year 2015) | Reply to the OECD Annual National Accounts Questionnaire from the Australian Bureau of Statistics, Canberra. The official estimates are published in 'Australian system of national accounts', ABS, catalogue 5204.0. | Reply to the OECD Annual National Accounts Questionnaire from Statistics Canada, Ottawa. The official estimates are published in 'National Income and Expenditure Accounts', Statistics Canada, catalogue13-201. | Data compiled by the 'Institut National de la Statistique et des Etudes Economiques (INSEE)', Paris and provided to the OECD by Eurostat. The official estimates are published in 'Comptes de la nation', INSEE. | Data compiled by the Centraal Bureau voor der Statistiek (CBS), Voorburg and provided to the OECD by Eurostat. The official estimates are published in 'Nationale Rekeningen', CBS. | Reply to the OECD Annual National Accounts Questionnaire from the United States Department of Commerce, Bureau of Economic Analysis (BEA), Washington. The official estimates are published in the 'Survey of Current Business', BEA. |
| AIC |  | Annual National Accounts of OECD countries. Final consumption expenditure at current prices and at constant prices (base year 2015). |  |  |  |  |  |
| Health consumption | Government FCE | Annual National Accounts of OECD countries. Individual consumption expenditure of general government at current prices and at  constant prices (base year 2015). |  |  |  |  |  |
|  | Household FCE - health | Annual National Accounts of OECD countries. Final consumption expenditure of resident households on the territory and abroad - health at current prices and at  constant prices (base year 2015). |  |  |  |  |  |
